# Morphological characteristic analysis of unrecorded freshwater diatoms in Korea

**DOI:** 10.1186/s42649-025-00112-8

**Published:** 2025-05-17

**Authors:** Daeryul Kwon, Kyeong-Eun Yoo, Hyeonjin Cho, Sukmin Yun, Chaehong Park

**Affiliations:** 1 Nakdonggang National Institute of Biological Resources, Sangju, 37242 Republic of Korea; 2 Encountter the Ecology, Gwanggyojungang-ro 248, Yeongtonggu, Suwon, Gyeonggido Korea

**Keywords:** Newly recorded species, Diatom, Freshwater, Electron microscope

## Abstract

Diatoms are microalgae with a significant ecological role as primary producers, contributing approximately 20% of global carbon fixation. They exhibit remarkable evolutionary success and biodiversity, with approximately 18,000 species identified globally, including 2,323 species reported in Korea. In this study, we identified 13 unrecorded diatom species from various freshwater environments in Korea, including rivers, streams, reservoirs, and lagoons. Among these, the genera *Brachysira*, *Gomphonella*, and *Staurophora* were recorded for the first time in Korea, expanding the national species list. The unrecorded species included 12 pennate diatoms (*Adlafia multnomahii, Encyonema cespitosum, Placoneis symmetrica, Gomphonema incognitum, Rhoicosphenia baltica, Brachysira vitrea, Gomphonella fogedii, Hantzschia psammicola, Hyalosynedra laevigata, Pseudostaurosira parasitica, Tryblionella calida*, and *Staurophora amphioxys*) and one centric diatom (*Discostella lacuskarluki*). Morphological and ultrastructural analyses were conducted using scanning electron microscopy to characterize these species. This research highlights the biodiversity of Korean diatoms and their potential applications in environmental monitoring and nanotechnology, contributing to the broader understanding of diatom taxonomy and ecology.

## Introduction

Diatoms are microalgae that are widely distributed worldwide, especially in the freshwater environment of Korea, and appear as dominant species in the water layer in spring and fall (Robarts and Zohary 1987). Diatoms are important primary producers, accounting for ~ 20% of the carbon fixation of entire plants, and are considered highly successful creatures in terms of biological evolution and diversity (Field et al. 1998). They exist in single-cell or colony form and ~ 18,000 known species currently exist worldwide (Guiry and AlgaeBase 2024). Diatoms generally live in water, either in floating form or attached to substrates such as stones, sediments, and aquatic plants (Douglas 1958; Reid et al. 1995; Fenoglio et al. 2020). New species of diatoms are continuously being discovered in sediments in various forms, ranging from contemporary deposits to fossilized forms (Kwon et al. 2023), in accordance with the diverse habitats of these diatom species (Behrenfeld et al. 2021). As diatoms maintain their shape over a long period of time, they are indicative of temporal changes in the global environment (Kooistra et al. 2007). Owing to these characteristics, many recent studies have used diatoms to determine carbon storage or the nanoscale porous structure of diatoms (Lim et al. 2023). Unlike other microalgae classifications, diatoms can be identified according to their form, and they are continuously identified in Korea and globally, contributing to the development of the national species list (NIBR 2023; Kwon et al. 2021). Structurally, the diatom has a sturdy lid shape and is covered with either a frustule or a theca (Kröger and Wetherbee 2000). Diatoms consist of amorphous silica, which can be easily obtained from natural water (Lim et al. 2023). Diatoms are composed of silica and pennate diatoms are relatively heavy compared to centric diatoms, silicates tend to sink, which can utilize various inorganic nutrients in the lower aquatic layer (Smetacek 1985). Diatoms are largely classified into pennate and centric types (Barron 1987). Pennate diatoms have a left–right symmetrical structure and are generally shaped las an elongated lid (Kooistra et al. 2009). They are characterized by striae that extend vertically to both ends of the cell with parallel protruding veins, resulting in holes in the lid and forming a decorative pattern (Cox 2012). Pennate diatoms form monophilic groups known as Bacillariophyceae (Hejduková et al. 2019). Many epilithic diatoms belong to this category (Salomoni et al. 2006). In addition, some pennate diatoms have raphe, which allow streamlined movement and new habitat occupation (Ruck and Theriot 2011). Centric diatoms have a radial symmetrical structure and are characterized by an arrangement of striae that extend from the center to the edge of the plate (Ross and Sims 1972). Five unrecorded diatoms species (*Adlafia multnomahii, Encyonema cespitosum, Placoneis symmetrica, Gomphonema incognitum,* and *Rhoicosphenia baltica*) were found in rivers, five unrecorded diatoms (*Brachysira vitrea, Gomphonella fogedii, Discostella lacuskarluki, Hantzschia psammicola,* and *Hyalosynedra laevigata*) were found in a stream, two unrecorded diatoms species (*Pseudostaurosira parasitica* and *Tryblionella calida*) were found in reservoirs, and *Staurophora amphioxys* was found in lagoons. The genera *Brachysira*, *Gomphonella*, and *Staurophora* were reported for the first time in Korea, contributing to the national species list. Currently, 135 species of *Brachysira*, 43 species of *Gomponella*, and 18 species of *Stauropora* have been recorded globally. Therefore, the unrecorded diatoms excavated in this study were subjected to morphological and ultrastructure analyses using scanning electron microscopy. Of the 13 unrecorded diatoms excavated in this study, 12 were pennate diatoms and 1 was a centric diatom.

## Materials and methods

### Sampling sites and methods

Samples were collected from various freshwater environments in Korea. The collection period spanned from 2020 to 2024, during which samples were obtained at different times. Samples were collected from Changnyeonghaman Weir (CW), Nakdan Weir (NW), Namhan River (NR), Mangtan Bridge (MB), Gyeongancheon (GC), Hamancheon (HC), Namchang Bridge (NB), Haminji (HM), Gyeongpoho (GP) (Table 1). Floating diatoms were collected using a phytoplankton net with a ≤ 10-μm mesh, and attachment and epilithic diatoms were directly collected from the waterfront by rubbing a soft brush on the substrate (stones or aquatic plants) to obtain the attached organic material. In addition, water temperature, pH, salinity, electrical conductivity, dissolved oxygen, and turbidity were measured using a portable water quality-measuring device (ProDDS, YSI, Yellow Springs, Ohio, USA) to elucidate the basic water environment of the collection site.

**Table 1:** Study area characteristics and environmental factors

Species	Sampling site	Type	Coordinate		Water temperature (℃)	pH	Conductivity (µs/cm^3^)	DO (mg/L)	Sal (ppt)
			**Longitude (N)**	**Latitude (E)**					
*Adlafia multnomahii*	CW (h)	River	33°21′22.62"	126°18′22.01"	12.9	7.2	100.0	7.6	0.09
*Encyonema cespitosum, Placoneis symmetrica*	NW (g)		36º16′59.26"	128º30′19.23"	5.4	11.5	89.0	11.3	0.12
*Gomphonema incognitum*	NR (b)		37º59′50.68"	127º53′1.91"	8.1	8.56	253.6	13.82	0.12
*Rhoicosphenia baltica*	MB (c)		34°54′5.71"	126°31′26.74"	21.6	8.27	319.1	10.87	0.15
*Brachysira vitrea,* *Gomphonella fogedii*	GC (a)	Stream	37º13′35.84"	127º12′44.14"	3.6	8.13	247.3	14.98	0.12
*Discostella lacuskarluki*	HC (i)		35°47′4.2"	128°16′40.8"	16.6	9.5	109.9	12.7	0.05
*Hantzschia psammicola, Hyalosynedra laevigata*	NB (d)		35º47′57.6"	129º18′45.8"	11.0	8.0	121.9	11.3	0.45
*Pseudostaurosira parasitica, Tryblionella calida*	HM (f)	Reservoir	35°53′04.05″	127°56′26.05″	18.6	7.2	254.7	5.9	0.14
*Staurophora amphioxys*	GP (e)	Lagoon	38°28′22.81"	128°26′22.81"	9.9	8.6	19,200	12.1	23.58

### Pretreatment for specific identification

Samples pretreated in the field were placed in a conical tube (SPL Life Science Co., Ltd., Pocheon, Korea), diluted to a ratio of 1:1:1 of sample: hydrochloric acid (HCl): potassium permanganate (KMnO_4_), and placed in a conical tube. The tube was placed into a beaker containing distilled water and then heated on a hot plate (SMHS-3, DAIHAN Scientific Wonju, Korea). The mixture was stirred until the color changed from purple to transparent, washed with distilled water, allowed to stand for 24 h, and the supernatant was removed. Samples were washed five times using this method to purify residual reagents and organic matter. The cover glass was placed on the electric heater. 1 mL of the acid-treated sample was dispensed, heated, and dried. Subsequently, one drop of mountmedia (Fujifilm Wako Pure Chemical, Osaka, Japan) was added to the cover glass, covered, and the sealing agent was spread evenly throughout the sample. The cover glass was sufficiently pressed to prevent bubbles. Subsequently, it was sealed using transparent nail polish.

### Shape and microstructure analysis of unrecorded diatoms

Field emission scanning electron microscopy (Fe-SEM, MIR-3, Tescan, Czech) was used to observe the ultrafine structure the diatom that is not visible under an optical microscope. To identify the species of the diatom, Lange-Bertalot & Krammer (Lange-Bertalot et al. 2000) was referenced, and the species name and classification system were organized according to Algaebase (https://www.algaebase.org/). Unrecorded species were determined by confirming both the name and synonyms from the Database of National Species List of Korea (NIBR 2023).

## Results

Phylum Heterokontophyta

Class Bacillariophyceae

Order Cymbellales

Family Anomoeoneidaceae

Genus *Adlafia*

*Adlafia multnomahii* Morales & Le (2005) (Morales and Le 2005)

### Original description

EA Morales, M Le, A new species of the diatom genus *Adlafia* (Bacillariophyceae) from the United States. Proc. Acad. Nat. Sci. Philadelphia 154, 149–154 (2005).

### Habitat environment

**Fig. 1:**
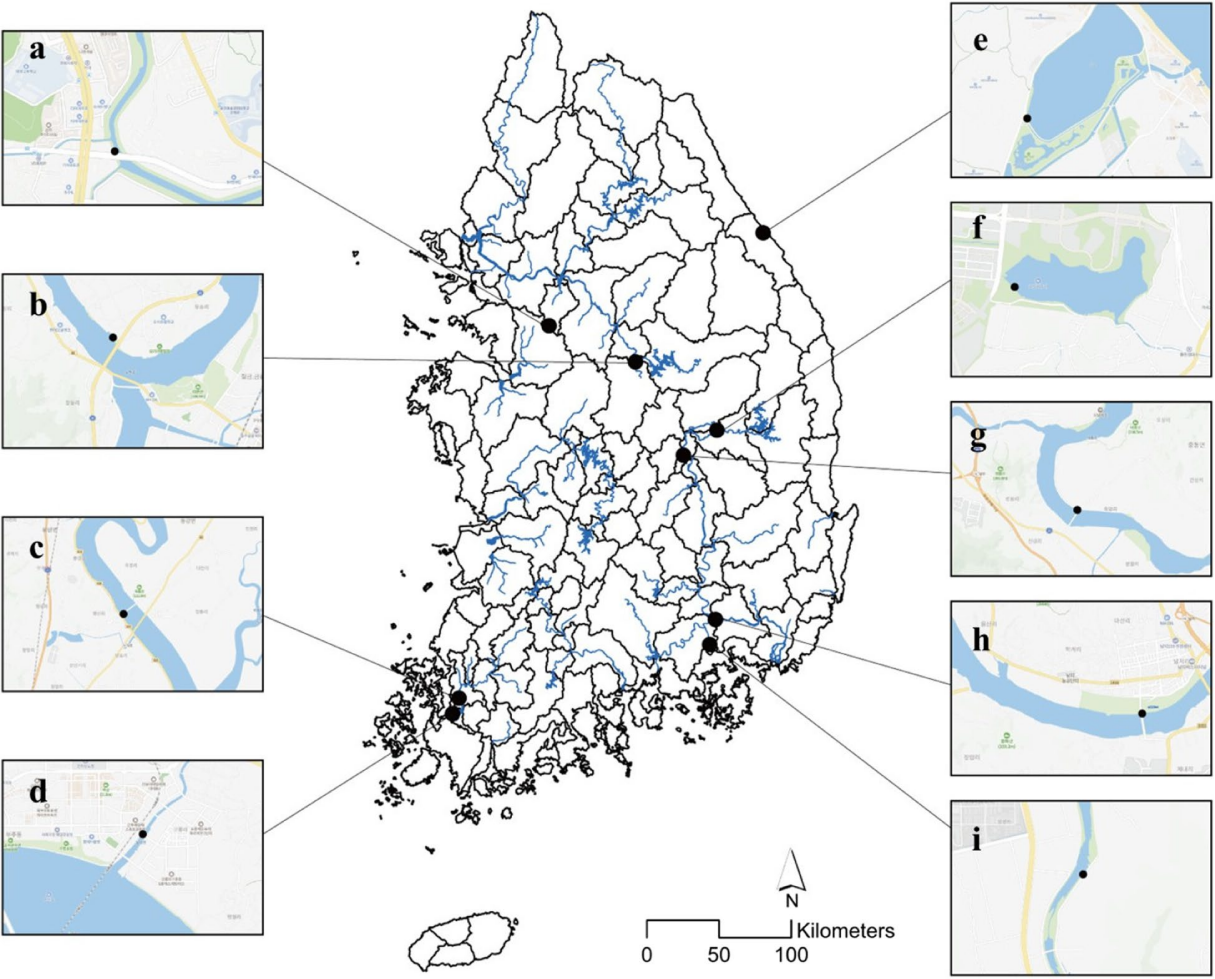
Diatom sampling site locations (**a** Gyeongancheon (GC); **b** Namhan river (NR); **c** Mangtan bridge (MB); **d** Namchang bridge (NC); **e** Gyeongpoho (GP); **f** Hominji (HM); **g** Nakdan weir (NW); **h** Changnyeonghaman weir (CW);_**i** Hamancheon (HC))

*Adlafia multnomahii* is a freshwater species that inhabits rivers in Oregon, California, Connecticut, New Hampshire, and Arkansas (Morales and Le 2005). It appears in neutral water (pH 7.1), at an electrical conductivity of 74–98.9 µs/cm^3^, alkalinity of 30 mg/L (CaCO_3_), phosphate concentration of 0.02–0.05 mg/L, nitrite + nitrate concentration of 0.11–0.651 mg/L, and temperature of 20.7–20.9 °C. In this study, it was observed in water characterized by neutral conditions (pH 7.4), water temperature of 24.1 °C, and electrical conductivity of 199.7 µs/cm^3^ and was discovered upstream from Changnyeonghaman weir. (Fig. 1h).

### Description

**Fig. 2:**
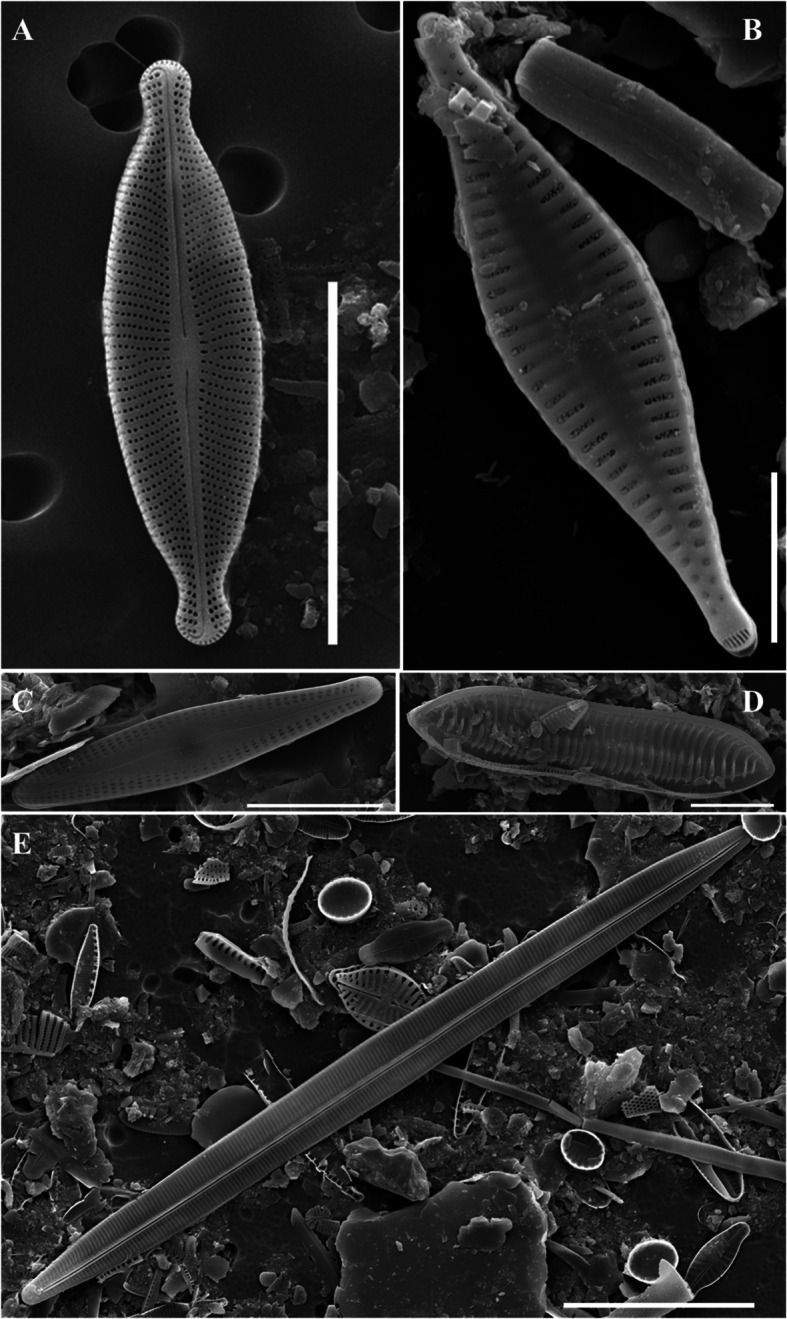
SEM microphotographs of Diatoms (valve view). **A** *Adlafia multnomahii*, **B** *Pseudostaurosira parasitica*, **C** *Gomphonema incognitum*, **D** *Tryblionella calida*, **E** *Hyalosynedra laevigata* (Scale bar: B = 5 µm, A, C, D = 10 µm, E = 20 µm)

The end of the needle-shaped valve is narrowed. Electron microscopy revealed that the raphe was well developed in a straight line, and both ends were sharply curved. The striae at the end of the valve were radially denser and curved toward the end, and other striae were strongly curved toward the central valve (Fig. 2a). This pattern of striae was not observed in a similar *Navicula* genus. It was 10–15 μm long, 3.5–4.5 μm wide, and had 35–40 μm striae in 10 μm.

Phylum Heterokontophyta

Class Bacillariophyceae

Order Fragilariales

Family Staurosiraceae

Genus *Pseudostaurosira*

*Pseudostaurosira parasitica* (W.Smith) Morales & Edlund (2003) (Morales and Edlund 2003)

*Basionym *Odontidium parasiticum Smith (1856)

### Original description

EA Morales, MB. Edlund, Studies in selected fragilarioid diatoms (Bacillariophyceae) from Lake Hovsgol, Mongolia. Phycol. Res. 51, 225–239 (2003). 

### Habitat environment

*Pseudostaurosira parasitica,* a brackish species reported in the United States of America Laurentian Great Lakes, is the most abundant species in Lakes Michigan and Huron and is widely distributed in coastal wetlands (Reavie and Kireta 2015). The source water had phosphorus and chloride concentrations of 30 µg/L and 10 µg/L, respectively. It also occurs in sediments from the River Quebec, Canada (Lavoie et al. 2008), and the St. Lawrence River (Reavie and Smol 1998). These taxa were observed in sediments from shallow lakes in Voyager National Park (Ek and Ryan Lakes), Lake Eil Royal National Park (Havy), and Otter Lake Lagoon in the Great Lakes National Park Network. In this study, it was found in Hominji was with a neutral pH (8.2), temperature of 4.9 °C, and electrical conductivity of 302.3 µs/cm^3 ^(Fig. 1f).

### Description

The genus *Pseudostaurosira* was generally chain-bound; therefore, the side valve surface was observed using optical microscopy. The valve was characterized by few striae (usually < four) and composed of an elliptical areola. *Pseudostaurosira parasitica* had a relatively wide valve center; however, the lanceolate narrowed strongly toward the end of the valve. The *pseudostaurosira* genus generally exhibit spines at the edge of the valve; however, *P. parasitica* did not have spines. The striae were located on the edge of the valve, and the long oval areola was regularly arranged on each side. Electron microscopy revealed pore fields at both ends of the valve (Fig. 2b). It was 9.5–18 μm long and 4–5 μm wide, with 19–21 striae in 10 μm.

Phylum Heterokontophyta

Class Bacillariophyceae

Order Cymbellales

Family Gomphonemataceae

Genus *Gomphonema*

*Gomphonema incognitum* Reichardt, Jüttner & Cox (2004) (Jüttner et al. 2004)

### Original description

I Jüttner, E Reichardt, EJ Cox, Taxonomy and ecology of some new *Gomphonema* species common in Himalayan streams. Diatom Res. 19, 235–264 (2004). 81 figs., 3 tables.

### Habitat environment

*Gomponema incognitum* is a freshwater species that originates from eastern Nepal and is heterogeneously distributed. It has been identified in samples from rivers in Georgia, North Carolina, and Alabama in the southeastern USA. All regions in the southeast are less nutritious and the least affected (Jüttner et al. 2004). In this study, samples were found in the mainstream of the Namhan River in water with a neutral pH (8.6), water temperature of 8.1 °C, and electrical conductivity of 253.6 µs/cm^3^ (Fig. 1b).

### Description

The valve exhibited a typical wedge shape. The headpole was blunt and wide, and the footpole, which had a pore field, was blunt and narrow. The central axial area of the valve was wide, and 2–4 striae were regularly arranged symmetrically on the left and right sides of the valve. The areolas were straight or curved C-shapes (Fig. 2c). The raphe was generally straight but was bent in the axial area. In addition, the raphe was connected to both ends of the valve but one end of the raphe was creased. One stigma was observed in the axial area. It was 17.8–29.5 μm long, 5–5.8 μm wide, and had 13–16 μm striae in 10 μm.

Phylum Heterokontophyta

Class Bacillariophyceae

Order Bacillariales

Family Bacillariaceae

Genus *Tryblionella*

*Tryblionella calida (Grunow)* Mann (1990) (Round 1990)

*Basionym* Nitzschia* calida* Grunow (1880)

### Original description

FE Round, The Diatoms: Biology and Morphology of the Genera, Vol. 747. Cambridge University Press (1990).

### Habitat environment

*Tryblionella calida* is a marine species that occurs in West Lake Okoboji and Lower Gar Lake in Dickinson County, Iowa, USA, carbonate-rich waters in Europe (Round 1990), and Mittersee lake, North Tyrol Limestone Alps, Austria (Round 1990). This species is commonly found in moderate to low nutrient conditions and in low abundance in eutrophic environments (Hofmann et al. 2011). In this study, it was observed in Hominji in water with neutral pH (8.2), water temperature of 4.9 °C, and electrical conductivity of 302.3 µs/cm^3^ (Fig. 1f).

### Description

The valve was generally linear, had a slightly concaved central portion, and was narrower toward the apices. The keel was very thick and strongly eccentric. The central nodule was located on the opposite side of the keel and was elongated around the valve. The number of fibulae was 7–9 in 10 μm. The transverse ribs were distinct, thick, linearly shaped, and slightly bent (Fig. 2d). It was 27–54 μm long, 8–10 μm wide, and had 30–32 μm striae in 10 μm.

Phylum Heterokontophyta

Class Bacillariophyceae

Order Licmophorales

Family Ulnariaceae

Genus *Hyalosynedra*

*Hyalosynedra laevigata* (Grunow) Williams & Round (1986)

*Basionym* Odontidium parasiticum Smith (1856).

### Original description

D.M. Williams, F.E. Round, Revision of the *Synedra* Ehrenberg genus. Diatom Res. 1, 313–339 (1986).

### Habitat environment

*Hyalosynedra laevigata* was first observed in the USA at Beck's Canal in West Okoboji Lake and in the Riverside Park Missouri River in Yankton, South Dakota. It was later observed at the bottom of a small pool in Silver Lake Fen, Iowa, and in rivers with moderate electrical conductivity and hardness throughout the USA (Hartley et al. 1986). It is also found in moderate alkaline, rich and poor, and rich rivers and lakes (Lange-Bertalot and Ulrich 2014). In this study, it was observed in the waters of Namchang Bridge in water with a neutral pH (7.9), water temperature of 21.5 °C, and electrical conductivity of 922.0 µs/cm^3^ (Fig. 1d).

### Description

*Hyalosynedra laevigata* exhibited an elongated, straight shape, with both ends of the valve rounded. Raphes forming long, continuous chain-like colonies were not observed. A weakly connected shape was observed, mainly through the ends of the valves. The margins were convex near the center and gradually converged almost linearly toward the apical region, where the curvature reversed, causing the sides to become either nearly parallel or faintly subcapitate. The sternum was narrow and located in the central valve of the cell. The striae exhibited left–right symmetry, a straight line, and were connected to the ends of the valves (Fig. 2e). It was 223–307 μm long, 5–8 μm wide, and had 26–28 striae in 10 μm.

Phylum Heterokontophyta

Class Mediophyceae

Order Stephanodiscales

Family Stephanodiscaceae

Genus *Discotella*

*Discostella lacuskarluki* (Manguin ex Kociolek & Reviers) Potapova, Aycock & Bogan (Potapova et al. 2020)

Basionym *Cyclotella lacus-karluki* Manguin ex Kociolek & Reviers 1996.

### Original description

MG Potapova, L Aycock, D Bogan, *Discostella lacuskarluki* (Manguin ex Kociolek & Reviers) comb. nov.: a common nanoplanktonic diatom of Arctic and boreal lakes. Diatom Res. 35, 55–62 (2020). doi:1080/0269249x.2020.1727569.

### Habitat environment

*Discostella lacuskarluki* is a freshwater species commonly found in the Arctic and Arctic lakes with a narrow geographical distribution. Its occurrence frequency in lakes across the northern hemisphere is increasing (Rϋhland et al. 2010), and it is widely distributed in tundra, Handae, alpine, and Agosan lakes across North America and Eurasia. However, this species may go unrecognized because Scanning Electron Microscopy (SEM) is required for accurate identification (Potapova et al. 2020). In this study, *D. lacuskarluki* was observed in the Hamancheon, characterized by neutral water (pH 7.9), a water temperature of 23.1 °C, and an electrical conductivity of 103.8 µs/cm^3^ (Fig. 1i).

### Description

**Fig. 3:**
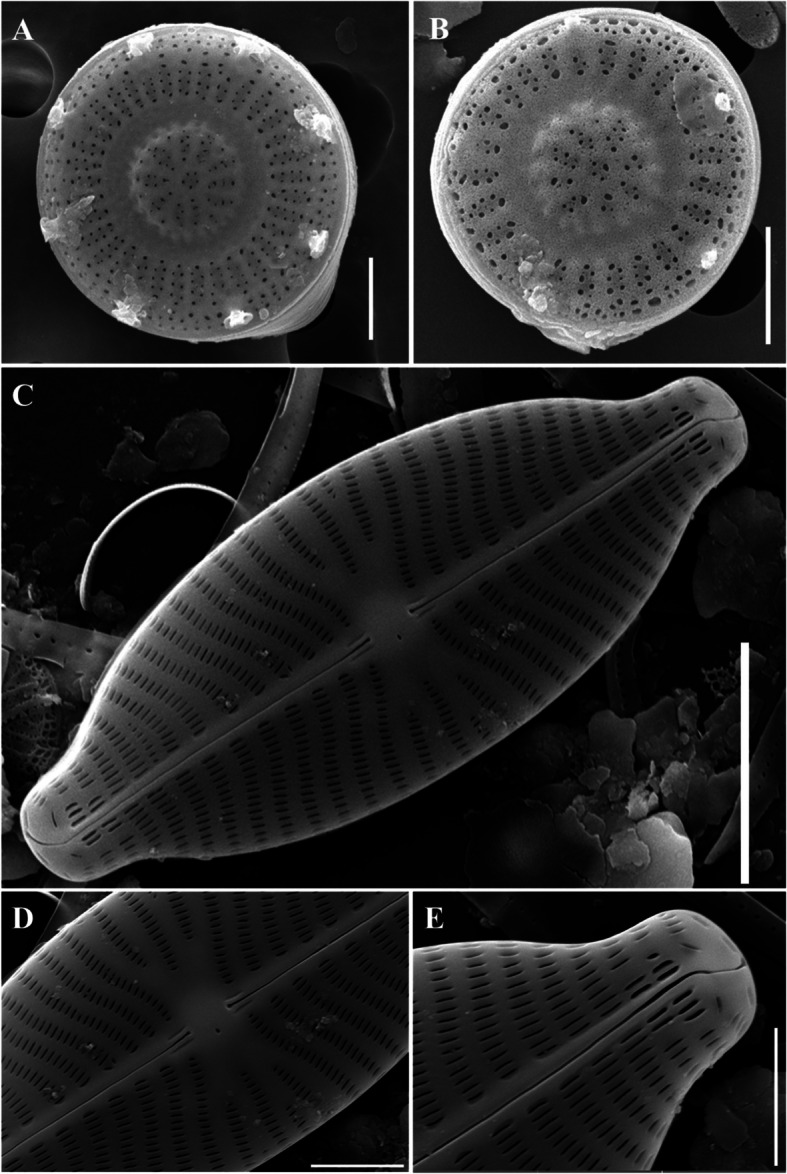
SEM microphotographs of Diatoms (valve view). A-B. *Discostella lacuskarluki* (Scale bar = 2 µm), C-E. *Placoneis symmetrica* D. External view of valve center. E. External view of head pole (Scale bar: D, E = 2 µm, C = 5 µm)

The cells were disk shaped and appeared individually. Stripes on the valve surface covered approximately 50–70% of the valve radius. The central area of the valve was convex, featuring ghost striae and rosettes, with 8–10 radially elongated striae located in the center. Ghost striae may also appear in valves with concave centers (Potapova et al. 2020). Approximately 5–8 rimoportula were positioned between the ribs at regular intervals along the edge of the valve (Fig. 3a–b). The valve diameter was 2.5–7 μm, and there were 13–19 striae in 10 μm.

Phylum Heterokontophyta

Class Bacillariophyceae

Order Cymbellales

Family Cymbellaceae

Genus *Placoneis*

*Placoneis symmetrica* (Hustedt) Lange-Bertalot (2005) (Metzeltin et al. 2005)

*Basionym Navicula constans var. symmetrica* Hustedt (1957).

### Original description

M D Metzeltin, H Lange-Bertalot, F García-Rodriguez, Diatoms of Uruguay. Compared with other taxa from South America and elsewhere. Iconographia Diatomologica 15, 1–736 (2005).

### Habitat environment

*Placoneis symmetrica* is a freshwater species found in Beck's Canal and Dickinson Co. Silver Lake, Iowa, USA. Cox & Mereschkowsky (Cox 2003) reported their presence in eutrophic freshwater areas. In this study, *P. asymmetrica* was observed upstream of Nakdan weir, characterized by neutral water (pH 8.9), water temperature of 15 °C, and electrical conductivity of 259.9 µs/cm^3^ (Fig. 1g).

### Description

The valve exhibited a linear lanceolate shape with slightly convex margins (Fig. 3c). The apices were round and blunt. The proximal raphe was linear, narrow, and straight, with faint lateral flanking (Fig. 3d). In contrast, the distal raphe was strongly curved and deflected toward the opposite side (Fig. 3e). The striae were radial and gently curved near the central part of the valve, whereas they formed a left–right symmetrical arrangement in a straight line at the apices. The areola exhibited a linear structure and the stigma was present in the central area (Fig. 3c–e). It was 13–25 μm long, and 7–11 μm wide, and has 30–32 striae in 10 μm.

Phylum Heterokontophyta

Class Bacillariophyceae

Order Cymbellales

Family Cymbellales incertae sedis

Genus *Gomphonella*

*Gomphonella fogedii* Edlund et al. (Edlund et al. 2024)

### Original description

MB Edlund, SA Spaulding, IW Bishop, M Potapova, SS Lee, PC Furey, E Jovanovska, Diatoms of North America: Nomenclatural transfers within the Bacillariophyceae 1. Phytotaxa 266, 195–205 (2024).

### Habitat environment

*Gomponella foggedii* is a freshwater species observed throughout the northern USA. It has been observed in the Laurentian Great Lakes, Lake Kinault on the Olympic Peninsula in Washington (Patrick and Reimer 1975), and the Pacific Northwest (Bahls 2024). In this study, it was observed upstream of Gyeongancheon characterized by a neutral pH (8.1), water temperature of 3.6 °C, and electrical conductivity 247.3 µs/cm^3^ (Fig. 1a).

### Description

**Fig. 4:**
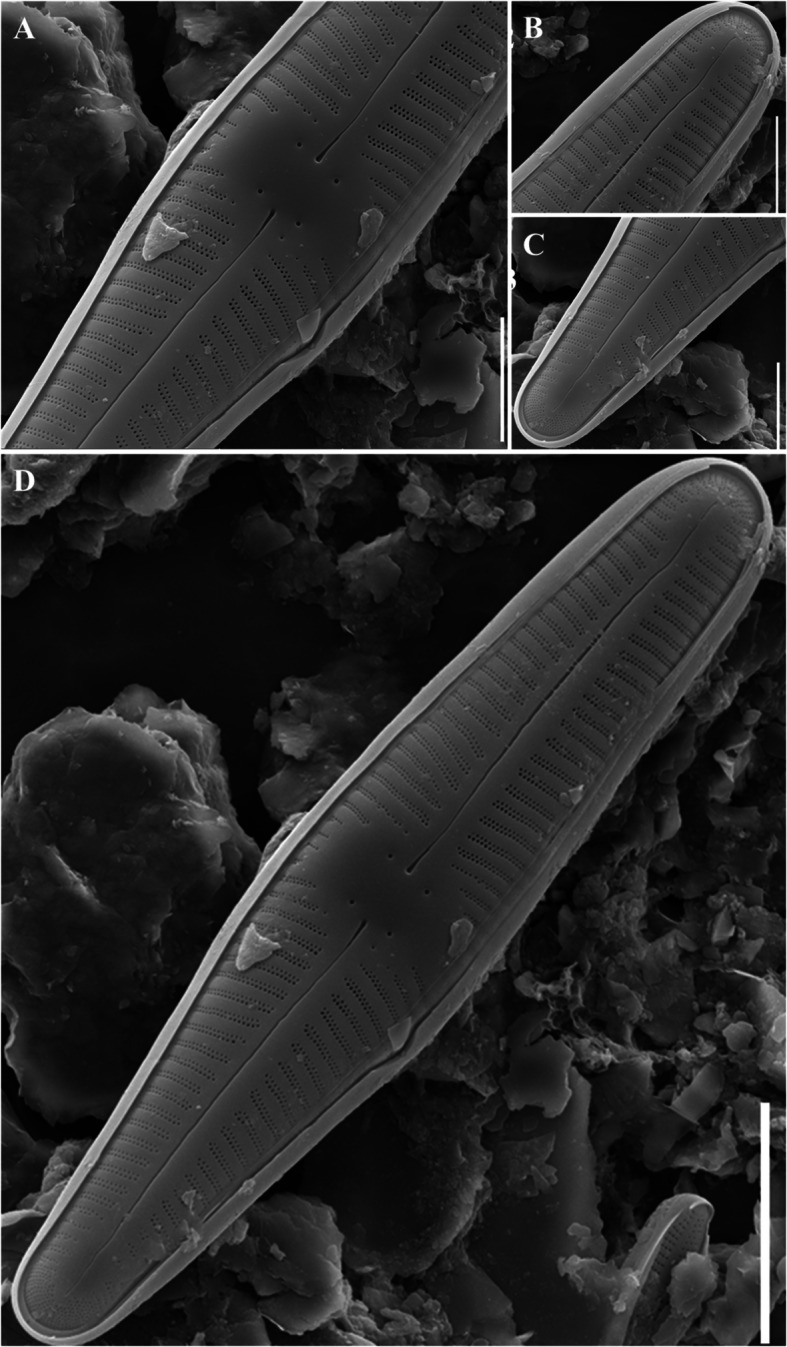
SEM microphotographs of *Gomphonella fogedii* (valve view). **A** External view of valve center. **B** External view of head pole. **C** External view of foot pole. **D** External view of valve (Scale bar: A–C = 5 µm, D = 10 µm)

The valve exhibited a wedge-like shape (Fig. 4d), whereas the head pole was rounded (Fig. 4b, d). Four stigmoids were observed in the central nodule and were arranged symmetrically along the left and right sides (Fig. 4a). Three to four short striae were observed at each edge in the center of the valve (Fig. 4a). The striae were weakly radiated and almost parallel to the head and foot poles. Electron microscopy confirmed that the striae were biseriate, a defining characteristic of *Gomphonella*. This feature was not visible with a standard light microscope. The raphe was straight and did not extend to the end of the valve (Fig. 4b–d). However, a pore field was observed at the terminal end of the raphe. It was 15–33 μm long, 5–6 μm wide, and had 16–18 striae in 10 μm.

Phylum Heterokontophyta

Class Bacillariophyceae

Order Cymbellales

Family Rhoicospheniaceae

Genus *Rhoicosphenia*

*Rhoicosphenia baltica* (Schumann) Levkov, Mihalić & Ector (2010) (Levkov et al. 2010)

*Basionym Rhoicosphenia fracta var. baltica* Schumann (1867).

### Original description

Z Levkov, K Caput Mihalić, L Ector, A taxonomical study of *Rhoicosphenia* Grunow (Bacillariophyceae) with a key for identification of selected taxa. Fottea 10, 145–200 (2010), 33 figs., 3 tables.

### Habitat environment

*Rhoicosphenia baltica* is a freshwater species found in several rivers in Southern California in the USA River Surface Waterfront Monitoring Program. These rivers ranged in pH (7.4–8.8), conductivity (112–2,325 µs/cm^3^), NO_3_ + NO_2_ concentration (0.006–0.048 mg/L), and phosphate concentration (0.0084–0.1400 mg/L) (Thomas and Kociolek 2015). In this study, it was found in the waters of the Mongtan Bridge, characterized by neutral water (pH 8.3), a water temperature of 21.6 °C, and an electrical conductivity of 319.1 µs/cm^3^ (Fig. 1c).

### Description

**Fig. 5:**
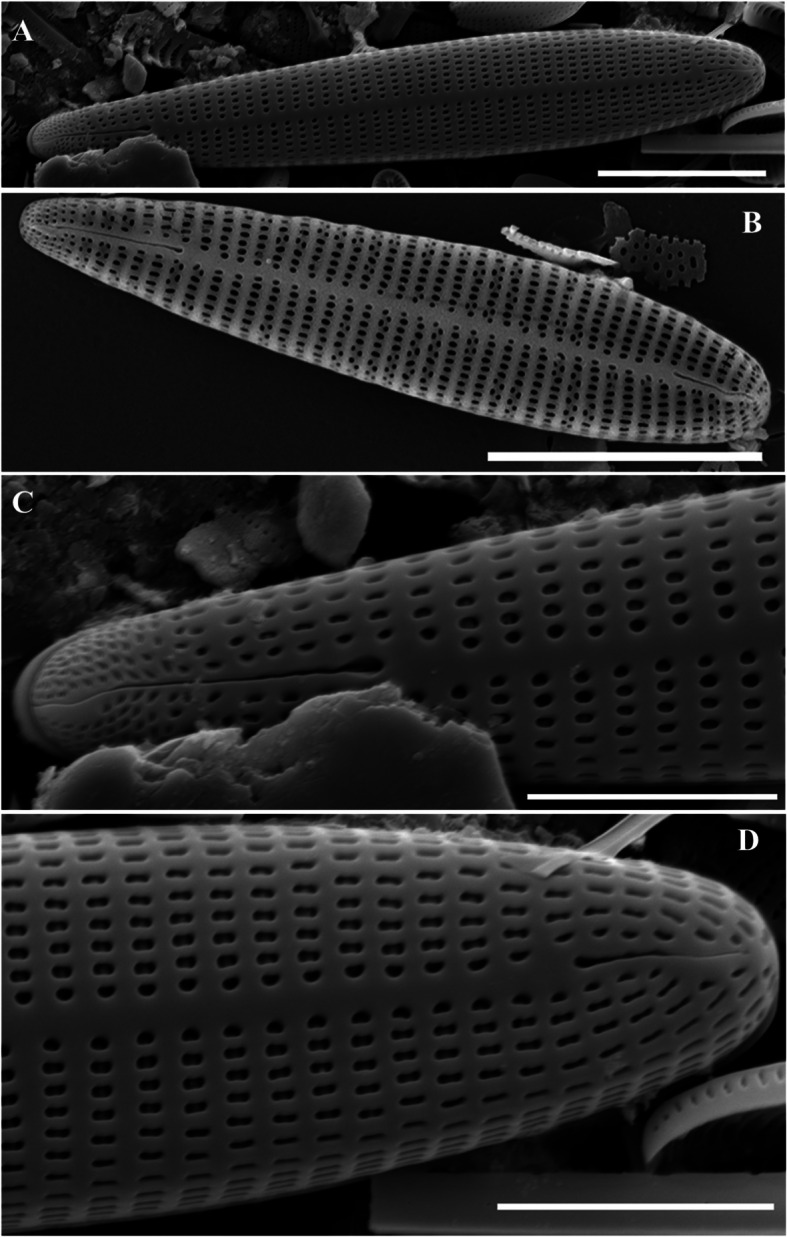
SEM microphotographs of *Rhoicosphenia baltica* (valve view) **A-B** External view of the valve. **C** External view of head pole. **D** External view of foot pole (Scale bar: C, D = 5 µm, A, B = 10 µm)

The valve was club shaped with rounded ends (Fig. 5a, b). The frustules were distantly archived (Fig. 5a, b). The raphe was not connected to the center of the valve and exhibited up-and-down asymmetry (Fig. 5c, d). The raphe extending up from the food pole was longer than that extending down from the head pole, and a pore field was observed in the food pole. The striae exhibited left and right symmetry and a radial shape at the end of the valve. The areola had a distinct oval shape (Fig. 5a–d). It was of 21–57 μm long, 4.5–8 μm wide, and had 15–18 striae in 10 μm.

Phylum Heterokontophyta

Class Bacillariophyceae

Order Cymbellales

Family Anomoeoneidaceae

Genus *Staurophora*

*Staurophora amphioxys*(W.Gregory) Mann (1990) (Round 1990)

Basionym Stauroneis amphioxys Gregory (1856).

### Original description

FE Round, The Diatoms: Biology and Morphology of the Genera, Vol. 747. Cambridge University Press (1990).

### Habitat environment

*Staurophora amphioxys* is a basal species that appears in deep and transparent brush lakes in northeastern Montana, USA, with an electrical conductivity of 5,760 µs/cm^3^, pH of 9.30, total nitrogen concentration of 1.3 mg/L, total phosphorus concentrations of 0.018 mg/L, dissolved oxygen of 7.50 mg/L, and water temperature of 18.0 °C. It is also widely distributed along the Atlantic coast of Europe and the Baltic Sea (Witkowski 2000) however, it has been reported to exist in the saltwater of the medium sea but that it is not widely distributed in the coastal areas of Europe (Hustedt 1959). In this study, it was found in Gyeongpoho, characterized by neutral water (pH 8.8), a water temperature of 2.6 °C, and an electrical conductivity of 38,203.0 µs/cm^3^ (Fig. 1e).

### Description

**Fig. 6:**
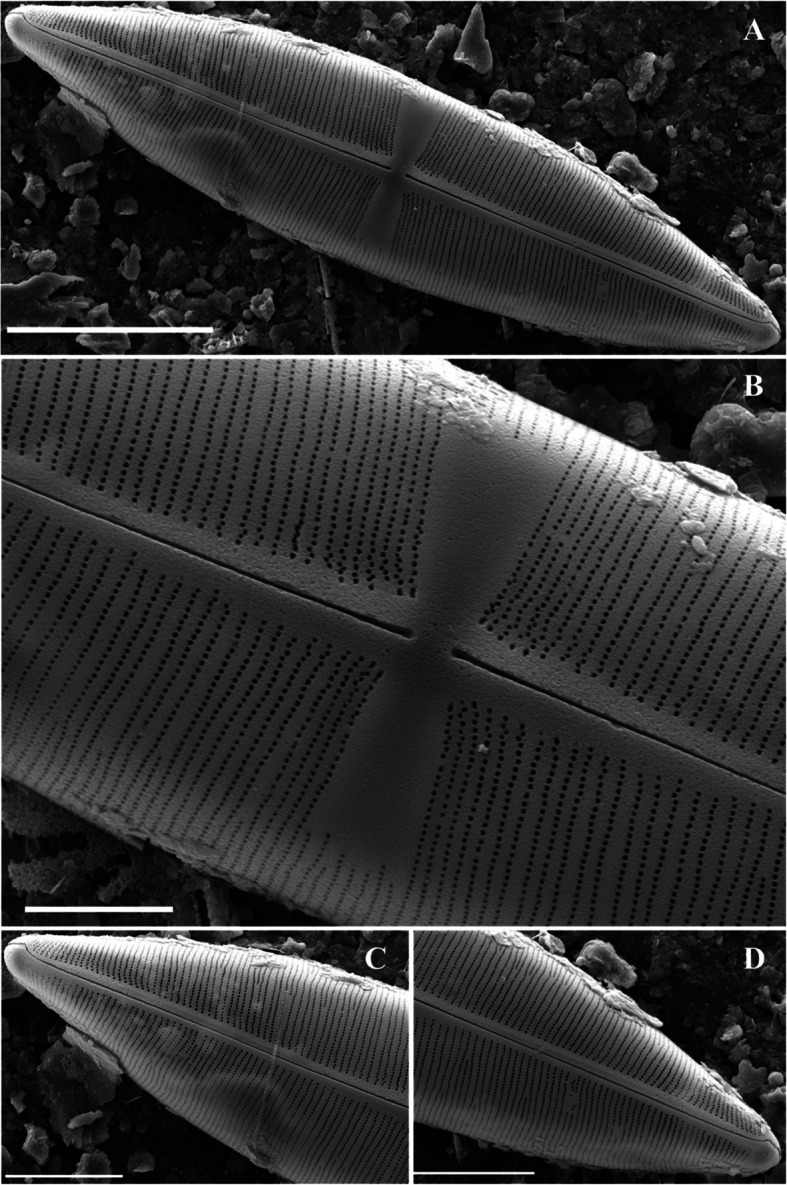
SEM microphotographs of *Staurophora amphioxys* (valve view). **A** External view of the valve. **B** External view of valve center. **C** External view of head pole. **D** External view of foot pole (Scale bar: B = 5 µm, C, D = 10 µm, A = 20 µm)

The valves exhibited a lanceolate shape, with distinctly curved valve faces seamlessly merging into a relatively deep mantle (Fig. 6a). Striae formed cross-like patterns. The fascia was present on both sides near the center of the valve and was interrupted by several short striae (7–8) near the edges (Fig. 6b). The striae consisted of small, round poroids (Fig. 6b). A raphe ran straight along the valve and connected to its ends, with slight bends observed at the terminal end (Fig. 6c, d). The central area of the valve featured a wide, transverse, bowtie-shaped fascia. The striae exhibited a radial arrangement, transitioning to a parallel pattern toward the ends of the valve, and were very dense (Fig. 6a–d). It was 40–67 μm long, and 12–17 μm wide, and had 13–16 striae in 10 μm.

Phylum Heterokontophyta

Class Bacillariophyceae

Order Naviculales

Family Brachysiraceae

Genus *Brachysira*

*Brachysira vitrea* (Grunow) Ross (1986)

*Basionym Gomphonema vitreum* Grunow (1878).

### Original description

B Hartley, R Ross, DM Williams, A check-list of the freshwater, brackish and marine diatoms of the British Isles and adjoining coastal waters. J. Mar. Biol. Assoc. U.K. 66, 531–610 (1986).

### Habitat environment

*Brachysira vitrea* occurs in the Cheyenne River, western South Dakota and Connelly Fen in the Great Plains, northwest Montana. Connelly Fen is characterized by a shallow and limestone environment with a pH of 7.6 and an electrical conductivity of 1,500 µs/cm^3^. *Brachysira vitrea* also occurs in lakes in Sweden and the southern Alps in rich and poor water dominated by lime (Lange-Bertalot et al. 1994). In this study, it was found upstream of Gyeongancheon in water with a neutral pH (8.1), water temperature of 3.6 °C, and electrical conductivity of 247.3 µs/cm^3 ^(Fig. 1a).

### Description

**Fig. 7:**
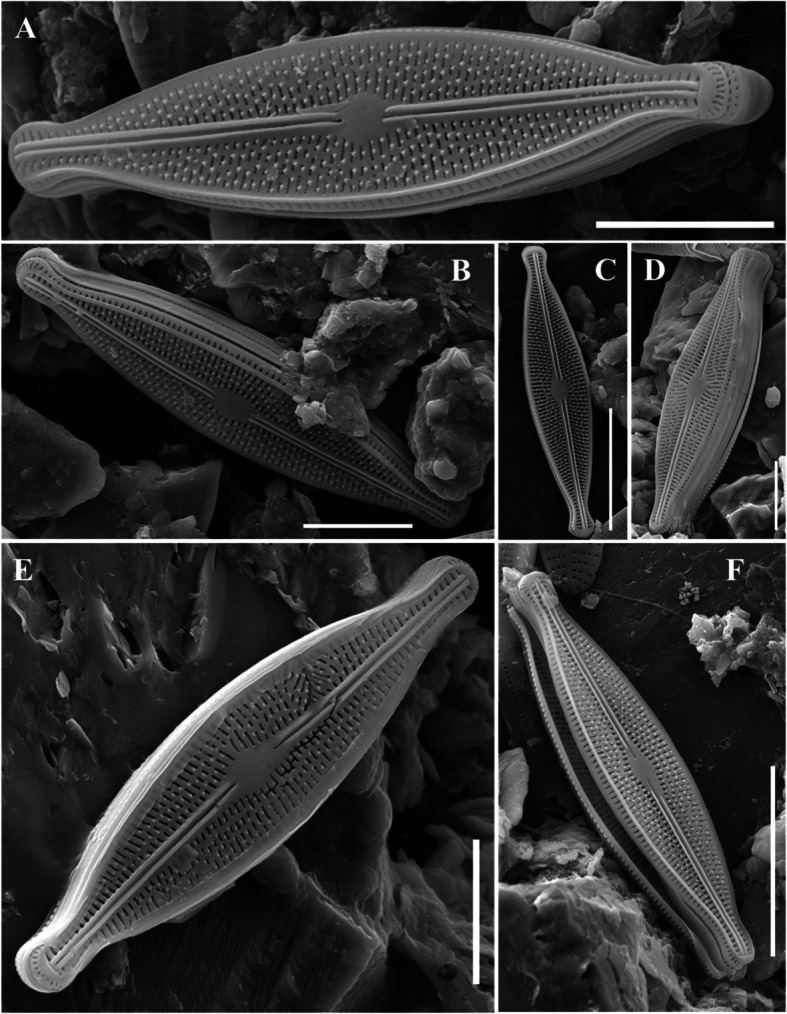
SEM microphotographs of *Brachysira vitrea* (valve view). **A-D** External view of the valve. **E** Internal view of whole valve (Scale bar: A, C, D, E = 5 µm, B, F = 10 µm)

Valves were linear to lanceolate, with rounded to protracted apices. The valves were generally symmetrical along the apical axis, although some valves exhibited asymmetry along the transapical axis. The striae were finely punctate, forming longitudinal undulations, whereas the raphe was straight with a narrow axial area. The striae were radiate and uniseriate, with irregularly spaced areolae creating undulating longitudinal lines (Fig. 7a–f). It was 12.4–25.4 μm long, 4.3–5.9 μm wide, and had 32–36 striae in 10 μm.

Phylum Heterokontophyta

Class Bacillariophyceae

Order Cymbellales

Family Encyonemataceae

Genus *Encyonema*

*Encyonema cespitosum* Kützing (1849) (Kützing 1849)

### Original description

FT Kützing, Species Algarum.-Lipsiae, Brockhaus (1849).

### Habitat environment

*Encyonema cespitosum* is a freshwater species distributed in poor- to moderate- nutrient water; however, it mainly appears in lakes and eutrophic water with intermediate conductivity. It rarely appears as benthic stones or plankton arranged in rows in mucinous tubes. In Korea, this species is rarely observed in the upstream and downstream sections of Chungju Lake. In this study, it was observed upstream of Nakdan weir characterized by neutral water (pH 8.9), a water temperature of 15 °C, and an electrical conductivity of 259.9 µs/cm^3^ (Fig. 1g).

### Description

**Fig. 8:**
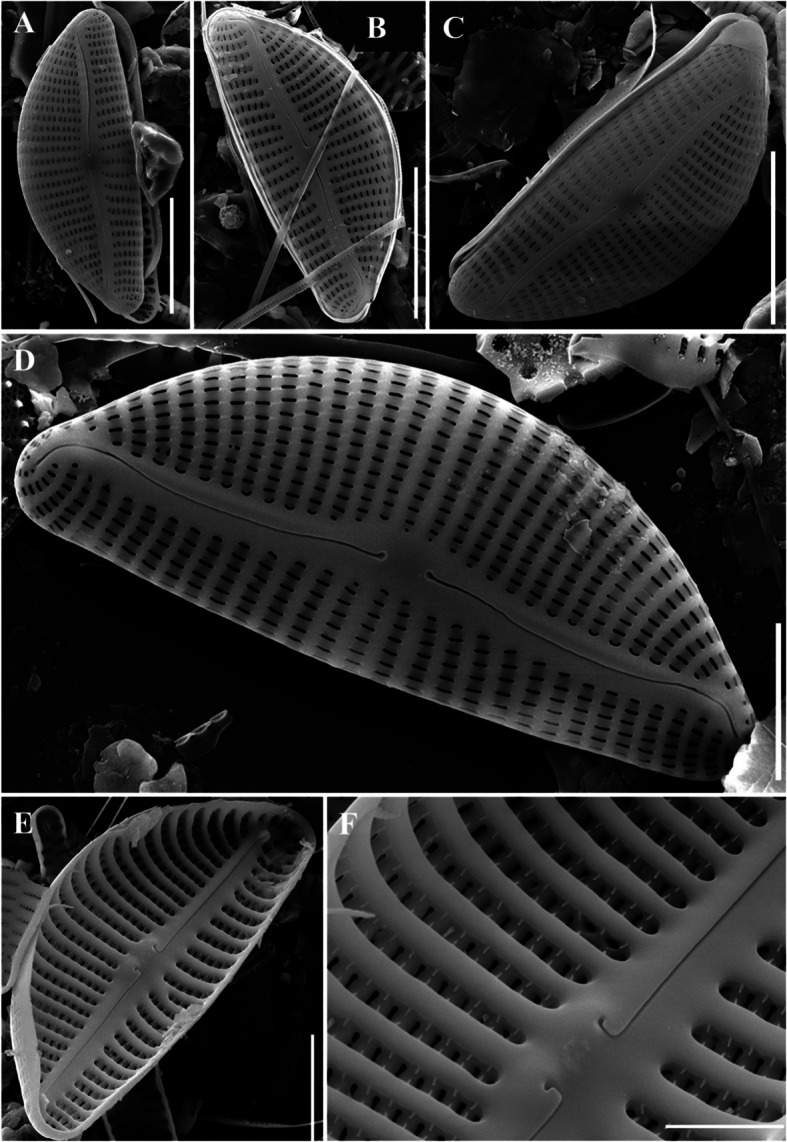
SEM microphotographs of *Encyonema cespitosum* (valve view). **A-D** External view of the valve. **E** Internal view of whole valve. **F** External view of valve center (Scale bar: F = 2 µm, D-E = 5 µm, A–C = 10 µm)

The valves were canoe-like or crescent-shaped (Fig. 8a–e). Frustules were asymmetrical along the apical axis but symmetrical along the transapical axis. The dorsal margin was strongly arched, whereas the ventral margin was straight or nearly straight (Fig. 8a–e). Valves were cymbelloid, with a convex dorsal margin, slightly convex ventral margin, and rounded or slightly protracted ends. The axial area was narrow and straight, and the raphe was either straight or slightly curved and displaced ventrally, with expanded proximal ends bent slightly dorsally and short-terminal fissures deflected ventrally (Fig. 8a–e). Striae slightly radiated and apically elongated, with some near the central area ending in small, round areolae (Fig. 8a–e). Apical pore fields were not observed. Vimines that separated the areolae were observed within the striae because of silica connections between the virgae. Additionally, the protrusions that delineated these vimines were regularly arranged on the back side of the valve (Fig. 8f). It was 18–58 μm long, 8–13 μm wide, and had 11–15 striae in 10 μm.

Phylum Heterokontophyta

Class Bacillariophyceae

Order Bacillariales

Family Bacillariaceae

Genus *Hantzschia*

*Hantzschia psammicola* Garcia-Baptista (1993) (Garcia-Baptista 1993)

### Original description

M Garcia-Baptista, Observations on the genus *Hantzschia Grunow* at a sandy beach in Rio Grande do Sul, Brazil. Diatom Res. 8, 31–43 (1993). 53 figs., 2 tables.

### Habitat environment

*Hantzschia psammicola* is a marine species reported (unverified) in relatively high numbers in streams and rivers in North America. A large population inhabits the soil and other aerobic habitats (Kwon et al. 2024). In this study, it was found in the waters of the Namchang Bridge, characterized by neutral water (pH 7.9), a water temperature of 21.5 °C, and an electrical conductivity of 922.0 µs/cm^3^ (Fig. 1d).

### Description

**Fig. 9:**
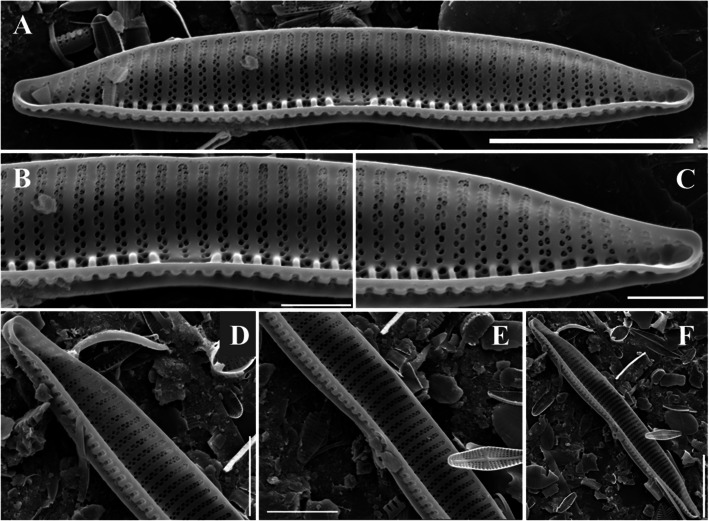
SEM microphotographs of *Hantzschia psammicola* (valve view). A, F. Internal view of the whole valve. B. External detail of the central area. C. Internal view of foot pole D, E. Internal valve viewed from the side (Scale bar: B–C = 5 µm, D-E = 10 µm, A, F = 20 µm)

The valve shape was linear lanceolate with tapering at both ends (Fig. 9a, f). The valve was asymmetrical along the apical axis, and the raphe was contained within the canal and eccentrically positioned on the valve margin. Light microscopy revealed the striae were dark linear lines; however, electron microscopy revealed that the areolae were arranged in a crossed pattern. The valve exhibited a stronger curvature toward the raphe (Fig. 9a–f). It was 50–61 μm long, 6–8 μm wide, and had 6–9 striae in 10 μm.

## Discussion

In this study, diatoms collected from seven sampling sites were identified through SEM. Among the 13 unrecorded diatom species, 5 were discovered in rivers, 5 in streams, 2 in reservoirs, and 1 in a lagoon. Unrecorded freshwater diatom species have been reported in Korea (Kwon et al. 2023, 2021; Kwon et al. 2024). Diatoms exhibit higher biodiversity and efficiency in species discovery than other microalgae classifications (NIBR 2023). The taxonomic classification of diatoms recently changed, with the phylum reassigned from Bacillariophyta to Heterokontophyta (Guiry et al. 2023). This shift suggests that significant changes may occur within lower taxonomic groups. In the present study, *Gomphonella*, *Staurophora*, and *Brachysira* were recorded for the first time in Korea, whereas *Adlafia*, *Hyalosynedra*, and *Rhoicosphenia* each had one previously reported species (NIBR 2023). These findings highlighted the importance of reporting on previously unrecorded diatoms. The genus *Gomphonella* is characterized by biseriate striae (Kwon et al. 2024), whereas *Gomphonema* has uniseriate striae (Ehrenberg 1832; Almeida et al. 2010). This difference leads to ambiguity in the genera *Gomphonella* and *Gomphonema* because they cannot be distinguished using optical microscopy (Jahn et al. 2019). However, the distinction was evident in *G. incognitum* and *G. fogedii*, making them suitable for comparative analysis (Fig. 2c and Fig. 4). Unlike *Gomphonema clevei* where they are absent, *G. incognitum* has C-shaped areolae (Kulikovskiy et al. 2020). This distinction can only be identified using SEM, making it challenging to differentiate between the two species using light microscopy. *H. psammicola* and *H. laevigata* typically inhabits marine environments (Belando et al. 2018); however, two species was observed in a freshwater habitat with low salinity (0.45‰, Table 1). Because the sampling site was near a brackish zone, some marine species likely infiltrated the freshwater environment. The genus *Discostella* includes species with diameters < 10 μm, making species identification challenging with light microscopy (Genkal 2015). Therefore, accurate identification requires SEM. The genus *Placoneis* was separated from *Navicula* in 1903 and is now classified under the family Cymbellaceae (Mereschkowsky 1903). A key feature of *Placoneis* is its uniseriate striae, composed of areolae forming a single row, whereas *Navicula* has lineate areolae-forming striae (Metzeltin et al. 2005). Despite this distinction, the distinction between *Placoneis* and *Navicula* is ambiguous (Mann and Stickle 1995; Kociolek and Thomas 2010). *Rhoicosphenia baltica* resembles *R. abbreviata* in valve morphology but has shorter raphae (Bahls 2024). The genus *Staurophora* has been compared to *Stauroneis*, with the latter possessing stauros and distinctly punctate striae(Vijver et al. 2005). In contrast, *Staurophora* features a fascia with short striae at the margins and striae composed of small round poroids that can be distinguished by light microscopy (Cocquyt and Staurophora caljonii spec. Nov. 2004). The genus *Brachysira* has not yet been reported in Korea and is characterized by wavy longitudinal striae with weak radial striae under light microscopy(Vijver et al. 2021). Currently, 135 species have been identified worldwide and many more are expected to be discovered in Korea in the future (Guiry and AlgaeBase 2024). *Encyonema* is often confused with *Cymbella*; however, *Encyonema* has ventrally deflected distal raphe ends, whereas those of *Cymbella* are dorsally deflected (Bahls 2007; Marquardt et al. 2017). Although visible under light microscopy, SEM provides a clearer differentiation. The genera *Hantzschia* and *Nitzschia* are often compared, where *Nitzschia* is generally characterized by valves with a linear lanceolate shape and *Hantzschia* is distinguished by bending and constriction at the central ventral margin of the valve (Maltsev et al. 2021). These features are distinguishable using both optical and electron microscopy. The discovery and documentation of species are crucial for securing biological sovereignty and enriching the list of national species. Additional unrecorded diatom species yet to be discovered in Korea, highlighting the need for continued exploration and reporting.

## Conclusions

This study identified 13 previously unrecorded diatom species from various freshwater environments in Korea, expanding the known diversity of diatoms in the region. Notably, the genera Brachysira, Gomphonella, and Staurophora were recorded for the first time in Korea, contributing to a more comprehensive national species inventory. The morphological and ultrastructural analyses conducted using scanning electron microscopy provided detailed insights into species characterization, facilitating accurate taxonomic classification. The findings of this study enhance our understanding of diatom biodiversity in Korean freshwater ecosystems and underscore the importance of continuous taxonomic surveys. Given the ecological significance of diatoms in primary production and environmental monitoring, future studies should focus on their functional roles in aquatic ecosystems and potential applications in nanotechnology and bioengineering. Moreover, further genetic analyses could complement morphological studies, providing a more robust framework for diatom taxonomy and phylogeny. By documenting new diatom species and their ultrastructural characteristics, this research contributes to global diatom biodiversity knowledge and supports future ecological and applied research endeavors.

## Data Availability

The datasets used and/or analyzed during the current study are available from the corresponding author on reasonable request.

## Abbreviations

SEM: Scanning electron microscope
CW: Changnyeonghaman Weir
NW: Nakdan Weir
NR: Namhan River
MB: Mangtan Bridge
GC: Gyeongancheon
HC: Hamancheon
NB: Namchang Bridge
HM: Hominji
GP: Gyeongpoho
